# Involvement of the HERV-derived cell-fusion inhibitor, suppressyn, in the fusion defects characteristic of the trisomy 21 placenta

**DOI:** 10.1038/s41598-022-14104-1

**Published:** 2022-06-22

**Authors:** Jun Sugimoto, Danny J. Schust, Tomomi Yamazaki, Yoshiki Kudo

**Affiliations:** 1 Department of Obstetrics and Gynecology, Hiroshima University, Hiroshima, Japan; 2 Department of Obstetrics, Gynecology and Women’s Health, University of Missouri School of Medicine, Columbia, MO USA

**Keywords:** Intrauterine growth, Mechanisms of disease, Endocrine reproductive disorders

## Abstract

Suppressyn (SUPYN) is the first host-cell encoded mammalian protein shown to inhibit cell–cell fusion. Its expression is restricted to the placenta, where it negatively regulates syncytia formation in villi. Since its chromosomal localization overlaps with the Down syndrome critical region and the TS21 placenta is characterized by delayed maturation of cytotrophoblast cells and reduced syncytialization, we hypothesized a potential link between changes in SUPYN expression and morphologic abnormalities in the TS21 placenta. Here we demonstrate that an increase in chromosomal copy number in the TS21 placenta is associated with: (1) reduced fusion of cytotrophoblast cells into syncytiotrophoblast in vivo, (2) increased SUPYN transcription, translation and secretion in vivo, (3) increased SUPYN/syncytin-1 receptor degradation in vivo, (4) increased SUPYN transcription and secretion ex vivo, (5) decreased cytotrophoblast cell fusion ex vivo, and (6) reciprocal response of changes in SUPYN and CGB in TS21 placental cells ex vivo*.* These data suggest direct links between immature placentation in Down syndrome and increased SUPYN. Finally, we report a significant increase in secreted SUPYN concentration in maternal serum in women with pregnancies affected by Down syndrome, suggesting that SUPYN may be useful as an alternate or additional diagnostic marker for this disease.

## Introduction

There are two main trophoblast subtypes in the hemochorial villous human placenta: cytotrophoblast cells (CTB) and the multinucleated syncytiotrophoblast (STB)^1–3^. Proliferative progenitor cytotrophoblast cells differentiate along two pathways. Some become invasive and leave the villous placenta as extravillous cytotrophoblast cells (EVT). EVT move along and across the maternal decidua, where they will encounter a unique set of maternal immune cells, invade and remodel the maternal spiral arteries and likely differentiate into the trophoblast giant cells that can be seen at the endometrial myometrial interface and in the inner third of the myometrium^4,5^. Other proliferative villous cytotrophoblast cells will leave the cell cycle and fuse to form villous STB, a major source of pregnancy specific hormones and the main site of maternal–fetal nutrient and gas exchange. Retroviral envelope proteins (env) are involved in fusion events in a diverse array of placental species^6–9^ and they have been shown to be required in humans and in mice for normal placental development^10–12^. In humans, the human endogenous retrovirus (HERV) protein, syncytin-1 (syn-1; aka *ERVW-1*) is necessary for the fusion of CTB into STB in the chorionic villus^10^. We have identified and reported on the presence of an endogenous inhibitor of cytotrophoblast cell fusion that is also derived from the env of an endogenous retrovirus^13–15^. Named suppressyn (SUPYN; aka *ERVH48-1*), this HERV-derived anti-fusogen is a 160 amino acid polypeptide that contains a stop codon upstream of the region encoding the transmembrane segment of an otherwise typical retroviral env^15^. This allows SUPYN to be detected in both cell-associated and secreted forms. SUPYN binds to the receptor for syn-1, the widely distributed neutral amino acid transporter, alanine serine cysteine transporter 2 (ASCT2, aka *SLC1A5*)^16,17^. Such binding likely occurs within the secretory pathway, alters the glycosylation of the receptor and inhibits the normal pro-fusogenic effects of syn-1/ASCT2 binding^14,15,18^. *ERVH48-1* colocalizes with the Down syndrome critical region on chromosome 21, of which there are three copies in affected Down syndrome patients^19^.

Trisomy 21 (Down syndrome; TS21) is the most common numerical chromosomal abnormality among live-born infants^20^. Placental defects in Down syndrome pregnancies have been well-described and appear to affect trophoblast cell differentiation, invasion and fusion^21–23^. Fusion defects have been the best studied. Morphologically, the villi of the TS21 placenta of the first and second trimester are characterized by a multilayered CTB component, which differs from age-matched disomic control placentas and placentas from other trisomic pregnancies^23^. Several investigators have shown defective fusion and abnormal pregnancy-specific hormone secretion for CTB that are isolated from TS21 placentas obtained across a wide range of gestational ages and allowed to differentiate in culture when compared to gestational age-matched counterparts^24–27^. We hypothesized that the overlap of the coding regions for SUPYN and the Down syndrome critical region on chr. 21 would result in augmented SUPYN dose effects in TS21 placentas and, in turn, decreased CTB fusion. In this paper, we show that abnormal expression of SUPYN is directly involved in immature placental formation in Down syndrome and that maternal serum SUPYN measurement may represent an additional or alternative marker protein for predicting fetal disease.

## Results

### Expression of SUPYN in the Down syndrome placenta

**Figure 1:**
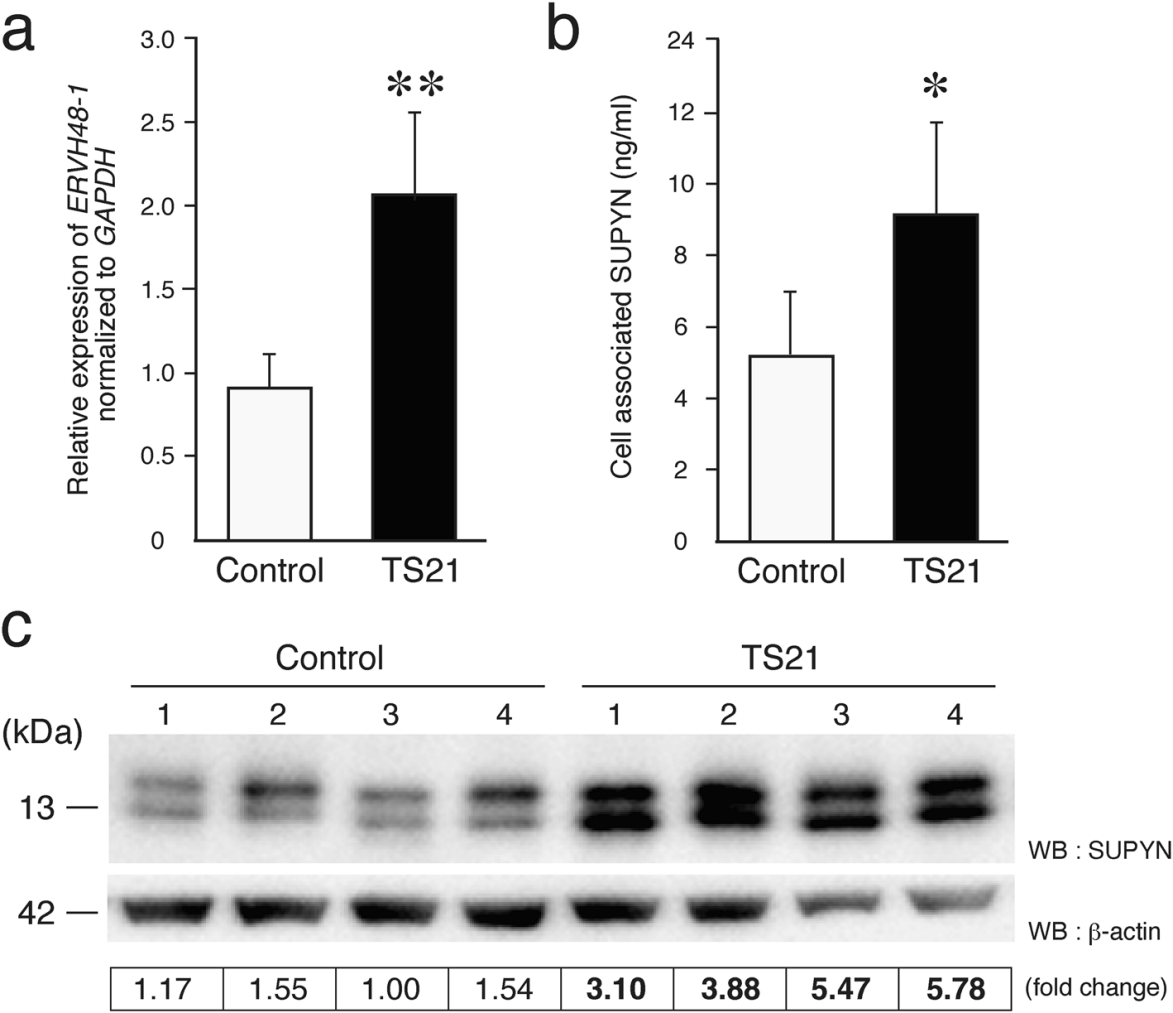
Expression of *ERVH48-1* transcripts and SUPYN protein in the TS21 placenta. (**a**) *ERVH48-1* gene transcripts in TS21 and gestational age-matched disomic control placental samples using quantitative RT-PCR. (**b**) SUPYN protein (ng/ml) levels in placental cell lysates from TS21 and gestational age-matched controls using a SUPYN-specific ELISA. Statistical analysis was performed using the Mann Whitney U test. Statistical significance was set at * : *p* < 0.05, ** : *p* < 0.01. Values represent mean ± SD (n > 5). (**c**) Western blot analyses using a monoclonal antibody against SUPYN. Lanes 1–4: four independent human placental samples from gestational age-matched control placentas ; Lane 5–8: four independent human TS21 placentas. Quantitative SUPYN protein levels were normalized to β-actin and presented in the bottom column.

Increased levels of *ERVH48-1* transcripts and SUPYN proteins were detected in Down syndrome placentas when compared to gestational age-matched disomic control placentas (16–20 weeks) (Supplemental Table S1). Using quantitative RT-PCR, a 2.2-fold increase in *ERVH48-1* transcript levels was detected in TS21 placentas when compared to controls (Fig. 1a). We have established a SUPYN-specific ELISA assay^14^ that can quantify cell-associated and secreted SUPYN protein in cell extracts and culture supernatants, respectively. We used this novel supplemental technique to validate an over two-fold increase in SUPYN protein levels in TS21 placental tissue extracts when compared to gestational age-matched controls (Fig. 1b). Western blotting analysis using an HRP-labeled SUPYN-specific monoclonal antibody confirmed increased normalized expression of SUPYN protein in TS21 placentas (Fig. 1c; Supplemental Figure S1).

Previous reports have described an increase in the number of CTB cells in the layer beneath the STB in TS21 placentas, suggesting a syncytialization defect^21,36^. We hypothesized that SUPYN expression would likewise be increased in the TS21 CTB cells when compared to disomic placental controls. Immunohistochemical staining of chromosome 21 trisomic and disomic age-matched control placentas for cytokeratin 7 (CK7) demonstrates previously reported morphologic changes in the TS21 placenta with increased numbers/layers of CTB cells noted in the TS21 villi when compared to disomic controls (Supplemental Figure S2). As expected, SUPYN protein co-localized with CK7 in the CTB layer and accordingly, the number of SUPYN-positive CTB cells increased in TS21 placentas when compared to chr. 21 disomic controls (Supplemental Figure S2). Further, in accordance with dosage effects, the level of SUPYN expression per CTB cell was also higher in the TS21 placenta (Supplemental Figure S2).

### Secreted SUPYN protein in maternal serum

**Figure 2:**
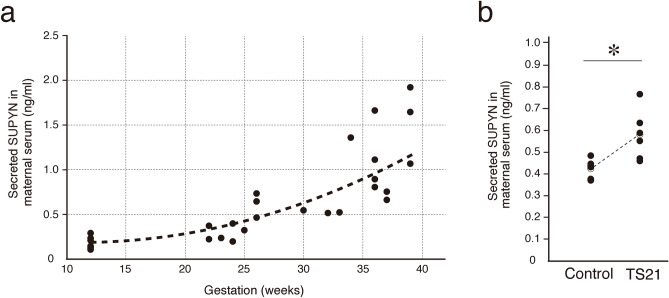
Quantification of secreted SUPYN protein in the TS21 maternal serum. (**a**) SUPYN-specific ELISA to monitor maternal SUPYN serum levels in normal pregnancies. (**b**) Quantification of secreted SUPYN protein (ng/ml) in serum from TS21 and gestational age-matched control pregnancies using a SUPYN-specific ELISA. Statistical analysis was performed using the Mann Whitney U test. Statistical significance was set at * : *p* < 0.05. Values represent mean ± SD (n > 5).

SUPYN is a secretory protein that can detected in maternal serum using a SUPYN-specific ELISA (Supplemental Figure S3). Levels of soluble SUPYN in maternal serum increased with increasing gestational age in normal pregnancies (0.2 ng/ml–2 ng/ml; Fig. 2a). When the same quantitative SUPYN ELISA was used to compare maternal serum from women at 16–20 weeks gestation with pregnancies affected by Down syndrome to gestational age-matched women with unaffected (disomic) pregnancies, we detected higher levels of SUPYN protein in the mothers carrying a baby with TS21 (Fig. 2b).

### Effects of SUPYN binding on ASCT2 glycosylation

**Figure 3:**
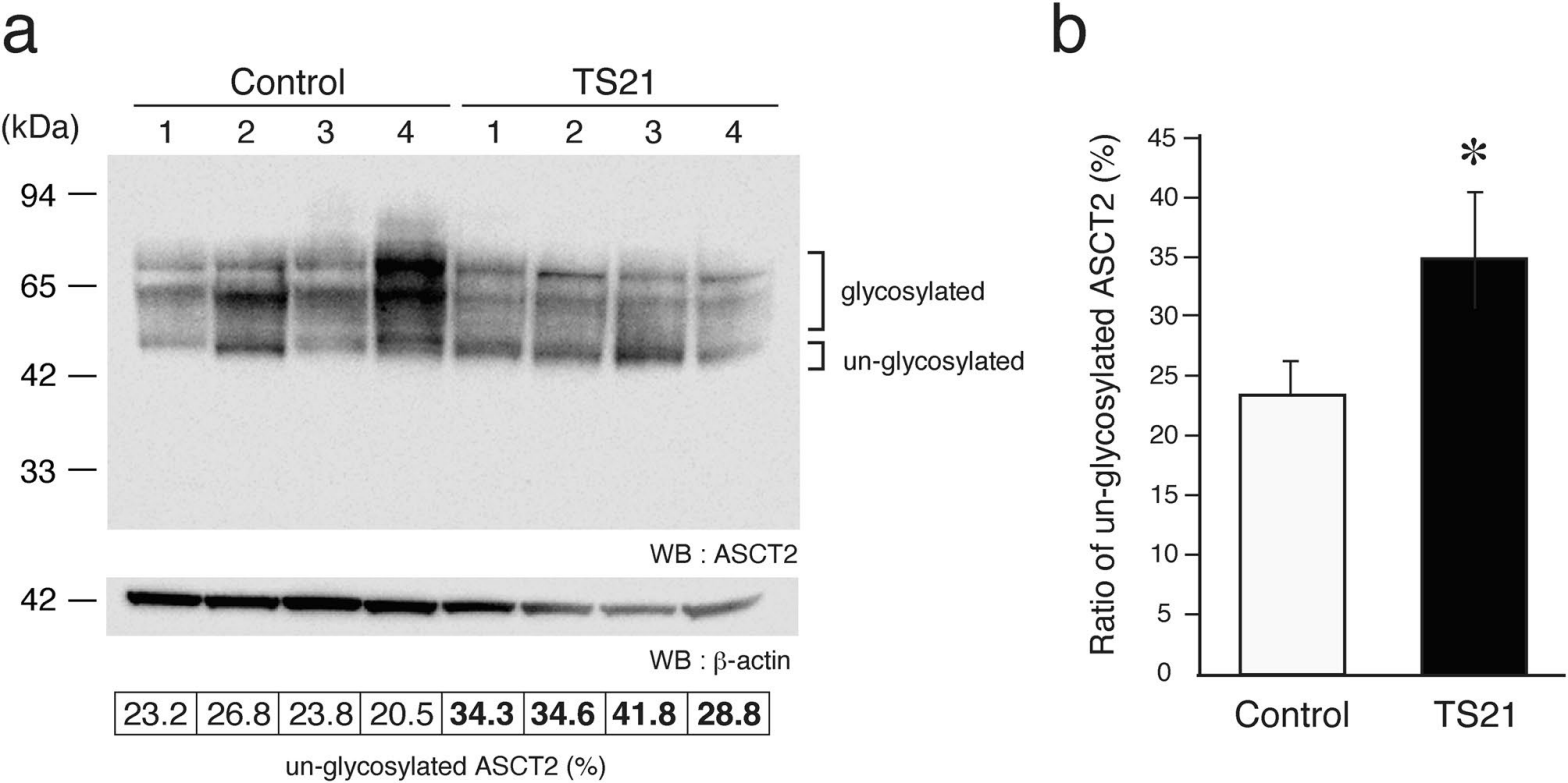
Association between SUPYN and ASCT2 induces glycosylation changes in TS21 placentas. (**a**) ASCT2 western blot analysis using gestational age-matched disomic control and TS21 placental samples. The numbers 1–4 represent sample replicates. Quantitative analysis of the percentage of ASCT2 in un-glycosylated bands when compared to total ASCT2 is presented at the bottom of the diagram. Glycosylated and un-glycosylated ASCT2 band intensities were quantified using CS analysis software from ATTO (2110030, Tokyo, JAPAN). The un-glycosylated ASCT2 ratio (%) represents the un-glycosylated band intensity / total band intensity × 100. (**b**) Average ratios combining all 4 replicates are graphically depicted. Statistical analysis was performed using the Mann Whitney U test. Statistical significance was set at * : *p* < 0.05. Values represent mean ± SD (n = 4).

The presence of SUPYN protein negatively regulates cell fusion by binding to the syn-1 receptor, ASCT2^14,15^. We previously reported that this binding occurs specifically inside of the cell, likely in the cytoplasm, and that SUPYN interaction with ASCT2 inhibits N-type glycosylation of ASCT2, likely while it is still in an immature state (“un-glycosylated”; molecular weight of about 49 kDa). We hypothesized that the overexpression of SUPYN protein in the TS21 placenta increases its level of association with ASCT2 and would result in an increase in the proportion of ASCT2 with immature N-glycosylation. We confirmed that N-glycosylation in TS21 placenta is aberrant. The un-glycosylated ASCT2 protein can be detected as a set of bands migrating at around 49 kDa (Fig. 3a, ASCT2u) and the partial or fully glycosylated forms of ASCT2 migrate at approximately 75 kDa. We detected an inversion of the 49 kDa:75 kDa, ASCT2u:ASCT2 ratio when comparing TS21 and disomic control placental samples. The ratio of un-glycosylated ASCT2 to glycosylated ASCT2 was 1.5 times higher in the TS21 placenta (Fig. 3b). In this experiment, a band of 13 kDa that reacts with the ASCT2 antibody was also noted. This protein, thought to be derived from the ASCT2 translation product, is also increased in the TS21 placentas when compared to disomic age-matched controls. Its function remains unclear (Supplemental figures S4).

### Ex vivo fusion analysis using primary TS21 and control CTB cells

**Figure 4:**
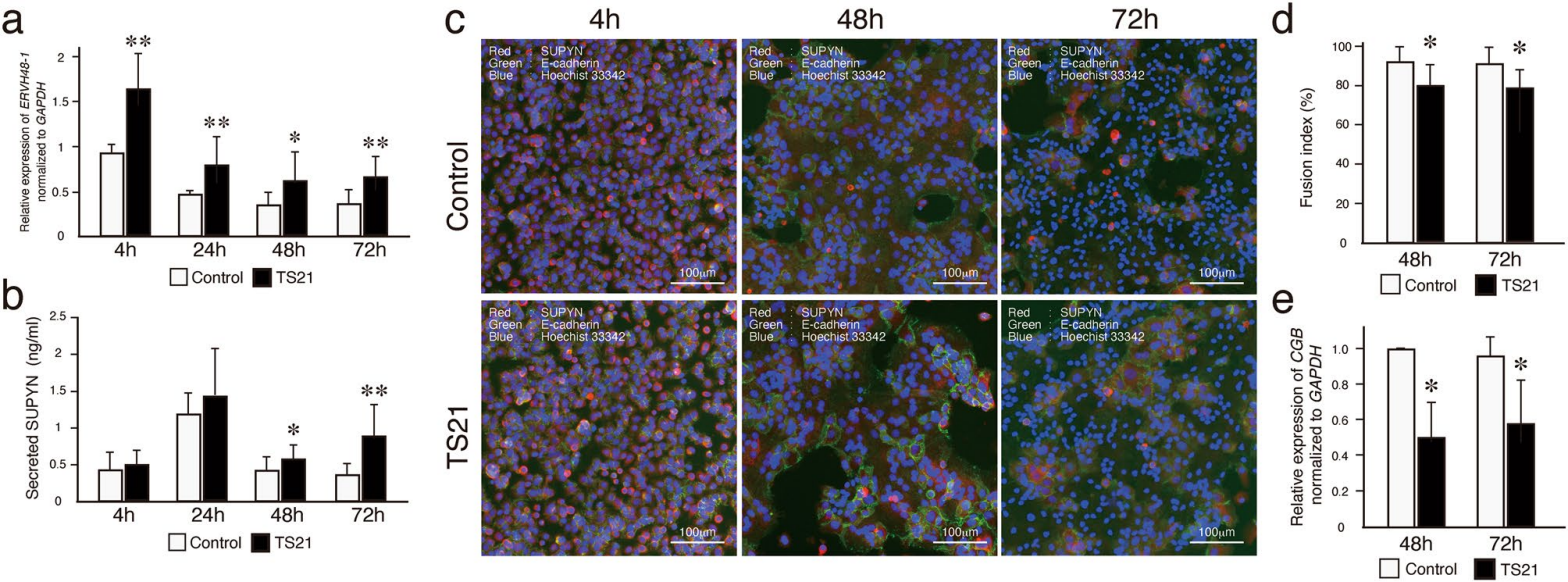
Expression of SUPYN and cell fusion indices in primary, spontaneously syncytializing human cytotrophoblast cells derived from TS21 and normal gestational age-matched control placentas. (**a**) *ERVH48-1* transcription in primary trophoblast cells was quantitated using RT-PCR. Fold-induction ratio of *ERVH48-1* transcripts in TS21 were compared with gestation matched control placenta at 4 h. (**b**) Detection of SUPYN protein in primary trophoblast cell culture supernatants by a SUPYN-specific ELISA (ng/ml). Experiments were performed in duplicate using 3 independent placental samples. Statistical analysis was performed using the Mann Whitney U test. Statistical significance was set at * : *p* < 0.05, ** : *p* < 0.01. Values represent mean ± SD (n = 3). (**c**) Time course of SUPYN protein expression in cultured primary, syncytializing cytotrophoblast cells from TS21 and gestational age-matched control placentas. Immunofluorescence images were obtained at 4 h, 48 h and 72 h after plating [SUPYN (red), E-cadherin (green) and Hoechst 33342 (blue)]. Upper panels are of representative cultured primary cells from gestational age-matched control placentas; lower panels are of representative cultured primary cells from TS21 placentas. Scales are as indicated. (**d**) The number of nuclei in SUPYN-expressing cells was counted and fusion indices were calculated. Experiments were performed using three independent samples with 5 independent microscopic fields evaluated for each sample. Values represent mean ± SD (n = 3). Statistical analysis was performed using the Mann Whitney U test. Statistical significance was set at * *p* < 0.05. (**e**) Expression of *CGB* transcripts from TS21 and gestational age-matched control placentas. Quantitative analysis of *CGB* gene expression in TS21 cells was compared to gestational age-matched control placental cells at each time point. Statistical analysis was performed using the Mann Whitney U test. Statistical significance was set at * *p* < 0.05.

Next, ex vivo functional analyses were undertaken using primary trophoblast cell culture to clarify whether the morphologic changes in CTB fusion seen in TS21 placentas in vivo was possibly dependent on the SUPYN protein also detected in these same cells in vivo. Isolated cytotrophoblast cells from TS21 and gestational age-matched control placentas were cultured for 72 h in media supporting CTB fusion in a 20% oxygen environment. *SUPYN* gene *(ERVH48-1)* expression was tracked across time in culture by quantitative RT-PCR as reported previously^14^. Normalized *ERVH48-1* gene transcripts were highest at 4 h after CTB isolation but continued to be detectable throughout the 72 h culture period in both TS21 and control samples (Fig. 4a). As measured by ELISA, secreted SUPYN protein levels in the culture supernatants of primary cultured cells were also detectable throughout the culture period for TS21 and control CTB cultures. Here, the 24 h values were the most elevated, which may reflect the high *ERVH48-1* transcriptional activity seen at 4 h (Fig. 4b). *ERVH48-1* transcripts were significantly higher in TS21 CTB than in control CTB at the 4, 24, 48 and 72 h timepoints (Fig. 4a) and secreted SUPYN protein levels were significantly higher in the TS21 CTB at 48 and 72 h of culture when compared to gestational age-matched control CTB (Fig. 4b). In contrast, transcript levels for genes encoding syncytin-2, which encodes the pro-fusogenic protein syn-2 (aka *ERVFRD-1*) and those encoding the syn-1/SUPYN co-receptor, ASCT2, in CTB cells did not change significantly over time in culture with the exception of a single value at 4 h for *ERVFRD-1* gene expression. Although gene transcripts for proteins associated with syncytialization (syn-1 and MFSD2: Major Facilitator Superfamily Domain Containing 2A) were decreased after 48 and 72 h of TS21 cell cultures, these same genes were unaffected in the un-fused CTB cells at 4 and 24 h (Supplementary Figure S5).

To assess potential functional effects of the increase in SUPYN seen in the TS21 CTB cultures, we assessed cell fusion into STB using standard cell fusion ratios. Immunohistochemical staining for E-cadherin allowed assessment of cell borders and Hoechst staining allowed counting of nuclei which together allow for the detection of single nuclei CTB and multinucleated STB (Fig. 4c). Cell fusion indices calculated at 48 and 72 h revealed significantly lower CTB cell fusion in cells derived from TS21 compared with those from disomic control placentas (Fig. 4d). Consistent with the decrease in cell fusion indices in primary TS21 trophoblast cells cultured ex vivo, *CGB* gene transcripts (encoding β-hCG) were also significantly decreased at 48 and 72 h. when compared to disomic controls (Fig. 4e).

### SUPYN siRNA treatment increases *CGB* gene transcript levels in TS21 cells

**Figure 5:**
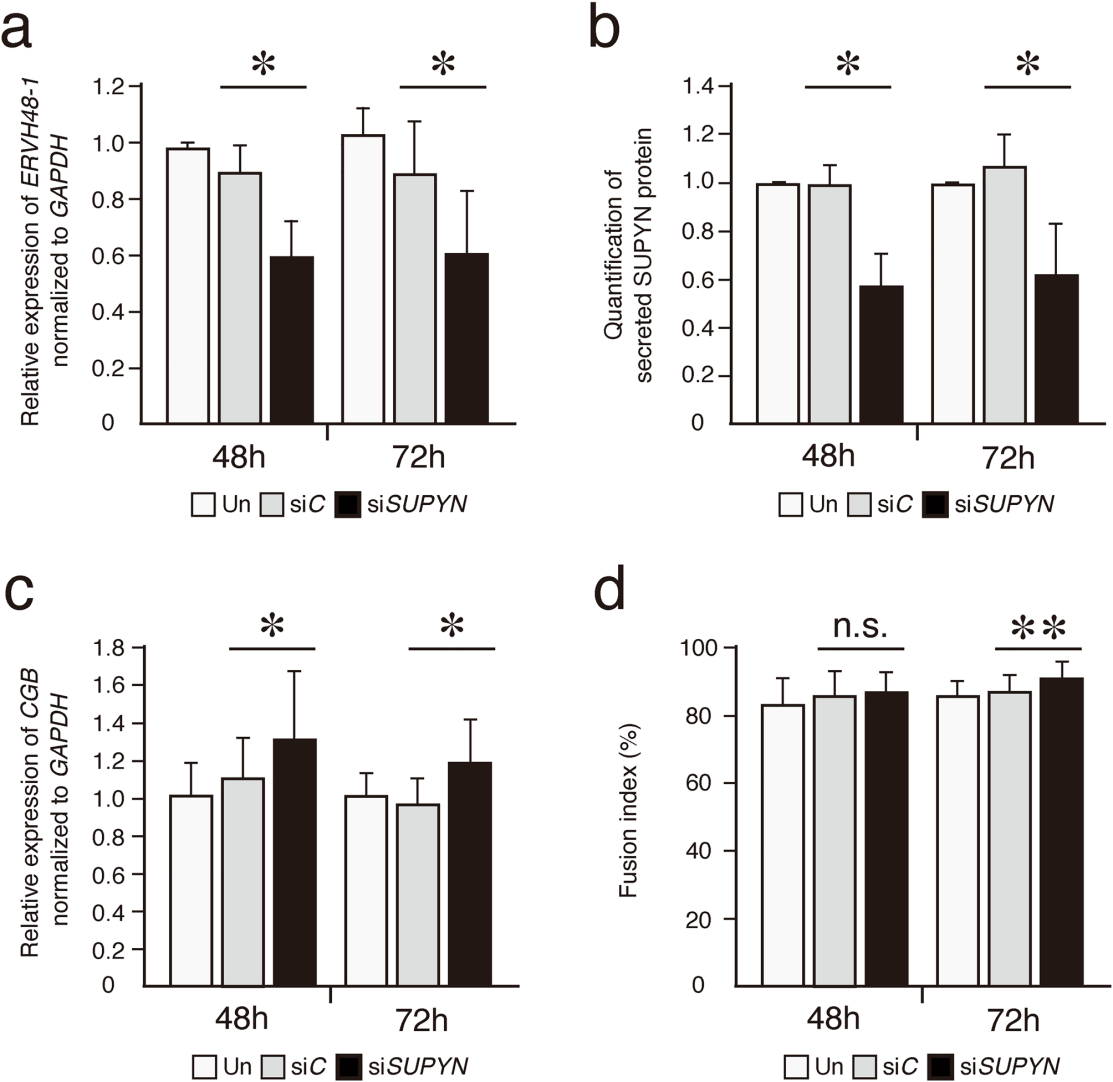
*SUPYN* knock-down increases *CGB* gene expression in TS21 primary cells. *SUPYN*-specific mRNA knock-down in TS21 primary cells using a siRNA (si*SUPYN*) or control siRNA (si*C*). (**a**) Quantitative RT-PCR results of *ERVH48-1* transcripts. (**b**) Secreted SUPYN protein in siRNA-treated and untreated cells. SUPYN protein levels in primary trophoblast cell culture supernatants were quantified by a SUPYN-specific ELISA (ng/ml). Quantification was normalized to untreated samples cultured for similar time periods (48 or 72H) (**c**) *SUPYN* knock-down induced an increase in *CGB* gene expression. Expression is normalized to untreated samples cultured for similar time periods (48 or 72H). (**d**) The number of nuclei in SUPYN-expressing cells was counted and fusion indices were calculated. Experiments were performed using four independent samples with 5 independent microscopic fields evaluated for each sample. Values represent mean ± SD (n = 4). Statistical significance was set at *p < 0.05 and **p < 0.01 compared to matched scrambled siRNA-treated samples using Mann–Whitney U-test. n.s. : not significant.

To demonstrate the specific anti-fusogenic effects of SUPYN in primary TS21 placental cells, cells were exposed to a SUPYN specific siRNA, and *CGB* gene transcript levels were assessed. *ERVH48-1* transcripts and SUPYN translation products decreased significantly after treatment with a SUPYN specific siRNA when compared with un-transfected and control, scrambled siRNA-treated samples (Fig. 5a, b). *CGB* transcripts were significantly increased at both 48 and 72 h (Fig. 5c). We also assessed cell fusion into STB using standard cell fusion ratios as in Fig. 4. Cell fusion indices calculated at 72 h revealed higher CTB cell fusion after treatment with the SUPYN specific siRNA when compared with scrambled siRNA-treated samples (Fig. 5d).During *SUPYN* knock-down experiments using human primary trophoblasts cells, limited knock-down efficiency results in robust fusion at baseline and it was challenging to see remarkable increase of fusion index.

## Discussion

The World Health Organization has estimated the incidence of Down syndrome is between 1:100 and 1:1100 live births and 3000–5000 children are born with this disorder in United States each year^28^. Approximately 6 million people worldwide have Down Syndrome^29^. It is the third most common trisomy detected in early pregnancy loss tissues using chromosomal microarray analyses^30^. Only a small number of people with Down syndrome are mosaics as 95% have the three copies of chromosome 21 in all cells. Essentially all who are personally affected by Down syndrome have neurodevelopmental challenges; over half will develop Alzheimer’s Disease in their 40 s and between 8 and 18% have autism^30–33^. Approximately 45% of Down syndrome babies (including stillbirths) will have congenital cardiac disease^31,34^.

We had a specific interest in the placental abnormalities associated with TS21. It has been reported that the number of unfused cytotrophoblast cells beneath the fused syncytiotrophoblast layer of the human placental villus is increased in the TS21 placenta^25,27,35,36^ and this morphology has been attributed to a CTB fusion defect^22,37–39^. We began our studies with the straightforward hypothesis that the dosage effect of an anti-fusogenic protein encoded by a retrovirus-derived sequence located in the q22.3 region of human chromosome 21, *ERVH48-1*^19^, was involved in this fusion defect. We have shown here, using primary tissues from TS21 and gestational age-matched control placentas, that *ERVH48-1* gene transcripts and SUPYN protein levels are indeed increased in the TS21 placenta in vivo. Confirming morphologic reports by others, we show that the number of CK7-positive CTB cells in the villi of placentas from TS21 pregnancies are increased and that these cells co-express the anti-fusogenic protein, SUPYN.

We extended these studies by using ex vivo analyses of primary cells from additional TS21 and gestational age-matched control placentas to validate tissue-level studies and examine functional outcomes. We confirmed that the levels of *ERVH48-1* transcripts and of SUPYN protein were higher in the TS21-derived primary CTB when compared with gestational age-matched control cells and that these differences were sustained throughout the 72 h culture period. In parallel analyses, no differences were noted for transcripts of other genes known to be involved in trophoblast fusion but located on chromosomes other than chromosome 21 at 4 and 24 h of culture with the exception of a single value at 4 h for *ERVFRD-1* gene expression. For example, *ERVW-1*, which encodes the pro-fusogenic endogenous retrovirus envelope protein syn-1, and that encoding the syn-1 and SUPYN receptor, ASCT2, are present on chr. 7q21.2 and chr. 19q13.32, respectively. *ERVFRD-1*, which also encodes the pro-fusogenic protein syn-2, and its receptor *MFSD2A*, are localized on chr. 6p24.2 and chr. 1p34.2, respectively. All of these transcript levels are similar in TS21 and control placenta-derived primary cells at 4 and 24 h except for *ERVFRD-1* gene expression at 4 h of culture. As predicted, levels of transcripts for genes associated with trophoblast syncytialization (*ERVW-1 and MSFD2A*) reflected the decreased level of fusion at 48 and 72 h in TS21 cells ex vivo. This suggests that an increased dose of *ERVH48-1* and SUPYN in the TS21 placenta may be primary drivers of morphologic changes in fusion. Further functional analyses in primary CTB cells cultured ex vivo show that CTB cell fusion and normalized transcripts for *CGB*, a characteristic placental hormone of the fused STB, are significantly lower in TS21 cell culture when compared to controls. When SUPYN levels were altered through *ERVH48-1*-specific knock-down^15^ in TS21 CTB cells cultured ex vivo, *CGB* gene expression and cell fusion could be recovered, demonstrating SUPYN-dependance of the effect and suggesting a potential modality for therapeutic intervention. Together, these results provide support for an important and direct role for the endogenous retrovirus-derived cell fusion inhibitor protein, SUPYN, in normal placental formation in humans. In severely affected placentas, abnormalities in placental development might be mechanistically linked to the high rate of early pregnancy loss in TS21 pregnancies. This hypothesis deserves further study.

Abnormalities in *ERVH48-1* dosage in TS21 may have implications in tissues other than the placenta in subjects with Down syndrome. Syn-1 and the syn-1/SUPYN receptor, ASCT2, are known to be expressed in the human brain^40–42^ and changes in syn-1, a closely-related *ERVW-1* envelope protein, MSRV, and ASCT2 have been linked to neurodegenerative disorders^41,43^ and schizophrenia^44^. It is certainly possible, therefore, that overexpression of cell-associated or secretory SUPYN in TS21 could alter syn-1 activities in the brain and could therefore be associated with the neurodevelopmental and neurodegenerative aspects of Down syndrome. *ERVH48-1* and SUPYN expression in the brain has not been reported in either normal or TS21 tissues and should be studied. Syn-1 is known to be expressed in smooth muscle cells and is involved in their fusion^45–47^. It is therefore also possible that *ERVH48-1* and SUPYN dosage could affect smooth muscle form and function in TS21, possibly including effects on the heart. More globally, a better understanding of tissue-specific control of trisomic gene expression, including the expression of *ERVH48-1*, may uncover other dosage related effects of this anti-fusogen. Future analyses of whole-body gene expression screening for Down syndrome patients are warranted.

Finally, we also include in this study results from a novel SUPYN-specific ELISA that can detect secretory SUPYN in maternal serum. We show that significantly higher levels of soluble SUPYN are detected in the sera of women pregnant with fetuses affected by TS21 when compared to gestational age-matched controls. Further, in maternal serum from normal pregnancies, secretion of soluble SUPYN increases in a gestational age-dependent manner. Since placental size, including its cytotrophoblast cell mass, increases throughout pregnancy, this may merely reflect placental growth and health; although this has not been formally tested in this study and future investigation is certainly warranted. Maternal serum screening for prenatal diagnosis of TS21 has historically included some combination of measurement of plasma protein A^48^, α-fetoprotein, β-hCG (human chorionic gonadotropin), non-conjugated estriol and inhibin^49^. Addition of SUPYN to these maternal serum analyte panels may increase accuracy and/or precision, sensitivity and/or specificity of screening results. Further analyses are warranted. In addition, should tissue specific SUPYN expression be ultimately linked to specific non-placental diseases in TS21, this SUPYN ELISA may be used in associated diagnostic testing.

In summary, we present data using a trisomic disease that an increased gene dosage for an endogenous viral envelope protein that inhibits cell fusion may play an important role in placental formation, reinforcing the concept that placental formation requires a fine-tuned and appropriately balanced mechanism to regulate trophoblast cell fusion. In this study, we show that increased *ERVH48-1* transcripts and SUPYN protein are associated with detrimental morphologic and functional changes in the TS21 placenta. Aberrant SUPYN levels could also be linked to other diseases of abnormal placentation, including hypertensive disorders of pregnancy, preeclampsia and fetal growth restriction. Further investigation is justified, including a pointed search for disorders of placentation that might be associated with decreases in SUPYN activity. These could include complete molar pregnancies, which have been characterized by syncytiotrophoblast hyperplasia, highly elevated maternal serum β-hCG levels and early onset pre-eclampsia ^50^.

## Methods

### Serum and placenta samples

Clinical samples were collected from otherwise discarded placentas from therapeutic terminations of pregnancy. All placentas were obtained from vaginal deliveries at 16–21 weeks of gestation. An amniocentesis or chorionic villus sampling procedure for genetic testing was performed between 15 and 18 weeks of pregnancy for suspected Down syndrome pregnancies and terminations of confirmed TS21 pregnancies occurred between 16 and 21 weeks. Age-matched disomic control samples were obtained from pregnancies affected by pPROM (preterm premature rupture of membrane) or from disomic pregnancies with other fetal malformations (see Supplemental Table S1). Maternal serum was obtained from these patients and/or healthy pregnant donors. All collections were approved by the Ethical Committee for Human Genome Research of the Hiroshima University (hi-222) and appropriate informed consent was obtained from all participants. All research was performed in accordance with relevant guidelines/regulations.

### Western immunoblotting

Twenty mcg of protein from placental samples was lysed in RIPA buffer (50 mM Tris–HCl, pH8.0, 150 mM Sodium Chloride, 0.5w/v% Sodium Deoxycholate, 0.1w/v% Sodium Dodecyl Sulfate, 1.0w/v% NP-40 substitute with protease inhibitor (165-26021: Fuji film, Osaka, Japan) and analyzed using standard PAGE and western immunoblotting. NuPAGE Bis–Tris 12% or 4–12% (for SUPYN and ASCT2, respectively) precast gels (NP0343, NW04127: Thermo Fisher Scientific, Waltham, MA, USA) were used for gel electrophoresis according to the instructions. Immunoblotting was performed by using the iBlot2 dry blotting system (IB21001, IB24002: Thermo Fisher scientific, Waltham, MA, USA) and HRP-labeled monoclonal anti-SUPYN antibody (1/2000 dilution) or polyclonal anti-ASCT2 antibody (1/1000 dilution, 8057: Cell signaling technology, MA, USA) were used for detection. The HRP (Horseradish peroxidase) signals were detected using a CCD imaging unit (Ez-Capture MG/ST; ATTO corporation, Tokyo, Japan).

### ELISA

A SUPYN-specific monoclonal antibody^14^ (clone: 2J16) was dissolved in carbonate buffer (0.5 µg/ml) and used as the capture antibody. 100 µl of the capture antibody solution was placed into wells on a 96-well plate and incubated overnight at 4 °C. Plates were washed 5 times with PBST (PBS with 0.05% Tween 20) using a microplate washer (ImmunoWash 1575:BioRad, Hercules, CA, USA) and blocked with 0.5% BSA / PBST for 1 h at room temperature. After washing, 50 μl of sample was diluted 1:2 with 25 μl of HAMA blocker (ab193969:abcam, Cambridge, UK) and 25 μl of PBS and dispensed into each well and incubated for 1 h at room temperature (RT). Plates were washed five times and HRP labeled anti-SUPYN monoclonal antibody (clone: 3H6) was used as a detection antibody at a concentration of 0.2 µg/ml. After 1 h at RT, plates were washed 5 times and incubated with 100 µl of POD (ELISA POD Substrate TMB kit: 05298-80: nakalai tesque, Kyoto, Japan) for 7 min at room temperature. Color development was stopped with 100 µl of H_2_SO_4_ and absorbance measured at a wavelength of 450 nm by a microplate reader (MPR-A100:ASONE, Osaka, Japan). ELISAs were performed in duplicate for each sample.

### Fluorescence immunocytochemistry

Primary cultured cells were fixed with 4% paraformaldehyde. Blocking was carried out with 5% normal serum/0.3% Triton X-100/PBS solution for 1 h at RT. The primary antibody reaction was allowed to proceed overnight at 4℃ with a combination of the polyclonal anti-suppressyn antibody (1/500 dilution) and the monoclonal anti-E-cadherin antibody (1/500 dilution : Abcam ab1414) in antibody dilution buffer (1% BSA/0.3% Triton X-100/PBS solution). Slides were then washed three times with PBS for 5 min each. Appropriate secondary Alexa Fluor 488 goat anti-mouse IgG or Alexa Fluor 555 goat anti-rabbit IgG antibodies (1/2000 dilution) were added for 1 h at RT. Nuclei were stained with Hoechst 33342 (0.5 μg/ml). Fluorescent signals were detected using a Fluorescence microscope unit (BZ-X710; KEYENCE Japan, Osaka, Japan).

### Manual quantification of fusion indices

Cell fusion was assessed using a Keyence microscope and quantitated using cell fusion indices. The multiple fluorescence images of 9 independent fields of a single well in a 48 well plate were captured (polyclonal SUPYN antibody: Red, monoclonal E-cadherin antibody: Green and Nucleus: Blue ) by a Keyence microscope (BZ-X710; KEYENCE Japan, Osaka, Japan). The fusion index quantitates the percentage of fusion events in a cell population. Analyses were performed using 5 randomly-selected independent fields in 3 (Fig. 4) or 4 (Fig. 5) independent samples. The number of fused syncytial aggregates and the number of nuclei in each aggregate was counted manually and fusion indices were defined as [(N-S)/T] × 100. N is the number of nuclei in syncytia, S is the number of syncytia, and T is the total number of nuclei counted.

### Statistical analysis

Statistical analysis was performed using the Mann Whitney U test. A *p* value below 0.05 (* : *p* < 0.05) or 0.01 (** : *p* < 0.01) was considered significantly different.

Detailed descriptions of experimental methods using standard approaches can be found in the supplementary materials and methods.

## Supplementary Information

Supplementary Information.

## Data Availability

The data that support the findings of this study are available from the corresponding author upon reasonable request.
