# Supplementary material for: Involvement of the HERV-derived cell-fusion inhibitor, suppressyn, in the fusion defects characteristic of the trisomy 21 placenta
Source: Sci Rep. 2022 Jun 22;12:10552. doi: 10.1038/s41598-022-14104-1 (PMC9218086; doi:10.1038/s41598-022-14104-1)
Supplement: Supplementary file 1 — Supplementary Information. [file 41598_2022_14104_MOESM1_ESM.pdf]

Involvement of the HERV-derived cell-fusion inhibitor, suppressyn, in the fusion defects characteristic of the trisomy 21 placenta

Jun Sugimoto[1,+,*], Danny J Schust[2,+], Tomomi Yamazaki[1], Yoshiki Kudo[1].

1. Department of Obstetrics and Gynecology, Hiroshima University, Hiroshima, Japan
2. Department of Obstetrics, Gynecology and Women's Health, University of Missouri School of Medicine, Columbia, MO USA

[*]Corresponding. jsokiaji1@gmail.com
[+]These authors contributed equally to this work

Supplementary Materials and Methods

(Immunohistochemistry)
Tissue blocks were prepared from formaldehyde-treated tissue using standard methods. Slides with 4 μm sections were heated at 60°C for 15 minutes and then standard deparaffinization with xylene was performed. Antigen retrieval was carried out using an electric kettle at 98°C for 40 minutes in 0.2 M citrate buffer at pH 6.0. Inactivation of endogenous peroxidase activity was performed in 0.3% H_2O_2/methanol at room temperature for 20 minutes. Sections were blocked with horse serum (for mouse antibodies) or donkey serum (for rabbit antibodies) at room temperature for 30 minutes. Primary antibodies were diluted as previously described[14] and sections were incubated with primary antibodies overnight at 4°C. Slides were washed three times in PBS and exposed to biotinylated antibodies at room temperature for 30 minutes, then to the streptavidin-HRP antibody at room temperature for an additional 30 minutes (VECTORSTAIN Elite ABC HRP kit : PK-6102, PK-6101:VECTOR Laboratories, Burlingame, CA, USA or Streptavidin-Biotin Complex Peroxidase kit: 30462-30：nakalai tesque, Kyoto, Japan). Color development utilized DAB (Peroxidase Stain DAB kit: 25985-50：nakalai tesque, Kyoto, Japan) and hematoxylin staining was done in the standard fashion at room temperature for 5 minutes.

(Isolation and culture of primary villous trophoblast cells)
Placentas were aseptically collected under an IRB-approved protocol and immediately cooled with iced PBS. Villous tissues were obtained from biopsies that were at least 5 mm from both the amniotic and decidual surfaces. The blood vessels were removed from the villous tissue and isolated villous tissues were minced and washed with cold PBS. This mixture was then filtered through a 100 μm stainless steel sieve and pretreated in a cell dissociation solution (Trypsin 0.125% (10×Trypsin:15090046: Thermo Fisher Scientific, Waltham, MA, USA), DNase I 200 U (DR-1S: Worthington, Lakewood, NJ, USA) /HBSS Ca+, Mg+ (14025: Thermo Fisher Scientific, Waltham, MA, USA) at room temperature for 5 minutes. The solution was again filtered through a 100 μm stainless steel sieve and approximately 10-20 g of wet volume tissue was placed in a 500 ml flask containing 50 ml of the same cell dissociation buffer with reciprocal shaking (90 rpm) at 37 °C. After one hour, the mixture was filtered through a 60 μm stainless steel sieve. Five ml of FBS was added to the solution and the extracted cells were recovered by centrifugation at 600 g×10 min. The cell pellet was suspended in Buffer 1 (0.1% BSA / DPBS) and layered on the top of a Percoll gradient (17089102: GE, Chicago, USA) layered from 70% to 10% at 10% intervals in a 15 ml tube and centrifuged at 1500 g×30 min. The target cytotrophoblast cell population was collected from each of the 40% and 50% layers and washed again with buffer 1. Isolated cells were counted, suspended at a concentration of 1×10^{6}/ml in Trophoblast Medium (TM) (#7121:Sciencell, Carlsbad, CA, USA) and cultured in aliquots of 200μl / well in 48 well plates at 37 °C in 5% CO_2. Four hours after seeding, the plates were manually agitated and nonadherent cells (e.g., hematopoietic cells) removed by changing the medium. Fresh media was added, and the cells were cultured for up to 72 hours at 37 °C, 5% CO_2 and 20%O_2, with daily media changes.

Supplementary Materials and Methods

(Transient gene knockdown by siRNA)

Isolated primary trophoblast cells were suspended at a concentration of 1×10^6/ml in Trophoblast Medium (TM) (#7121:Sciencell, Carlsbad, CA, USA) and cultured in aliquots of 200μl / well in 48 well plates at 37 °C in 5% CO_2. Four hours after seeding, the plates were manually agitated and nonadherent cells (e.g., hematopoietic cells) removed by changing the medium. Cells were transfected using 1 μl of RNAiMAX transfection reagent(13778100:Thermo Fisher Scientific, Waltham, MA, USA) and 10 pmol siRNA in 250 μl of Opti-MEM medium as previously described[15]. After 24 hours, medium was changed, and incubation continued for 72 hours with daily media changes.

(RT-PCR)

Beginning 4 hours after placement in culture, primary cytotrophoblast cells were collected every 24 hours. A Qiagen RNeasy Plus kit (74134: Qiagen, Valencia, CA, USA) was used for total RNA isolation in accordance with the manufacturer's instructions. 200ng of the total RNA was subjected to reverse transcription using a ReverTraAce kit (FSK-101:Takara, Shiga, Japan) at 30°C for 10 minutes, 42°C for 60 minutes and 95°C for 5 minutes.

(Quantitative RT-PCR)

Six microliters of a ten-fold diluted reverse transcription product was used for real time RT-PCR with SYBR Green I in 25μl total volume (Applied Biosystems *Power* SYBR Green Master Mix: 4368577: Thermo Fisher Scientific, Waltham, MA, USA) The SUPYN and other fusion-related genes primers used for real-time PCR have been previously described[14]. The analysis of relative gene expression data was performed using the $2^{-\Delta\Delta CT}$ method.

	Control	TS21
Gestation (weeks/day)	16w1d ~ 21w6d	16w3d ~ 21w2d
Placental weight (g)	126 ± 42.5	135 ± 80.1
Fetal weight (g)	250±100	259 ± 90.2
Fetal height (cm)	23.4±2.9	22.9± 3.6
Symptoms	pPROM, Acrania, Hydrocephalus, Amniotic band syndrome, Thanatophoric dysplasia, Renal agenesis.	Trisomy 21 diagnosed by amniocentesis. (Trisomy of chr. 21)
The numbers of samples	n = 10	n = 8

Supplementary Table S1. Detail of clinical samples.
There were no statistically-significant differences in gestational age, placenta weight, fetal weight or fetal height between gestational age-matched disomic control and TS21 samples using Mann Whitney U-testing ($p > 0.05$).

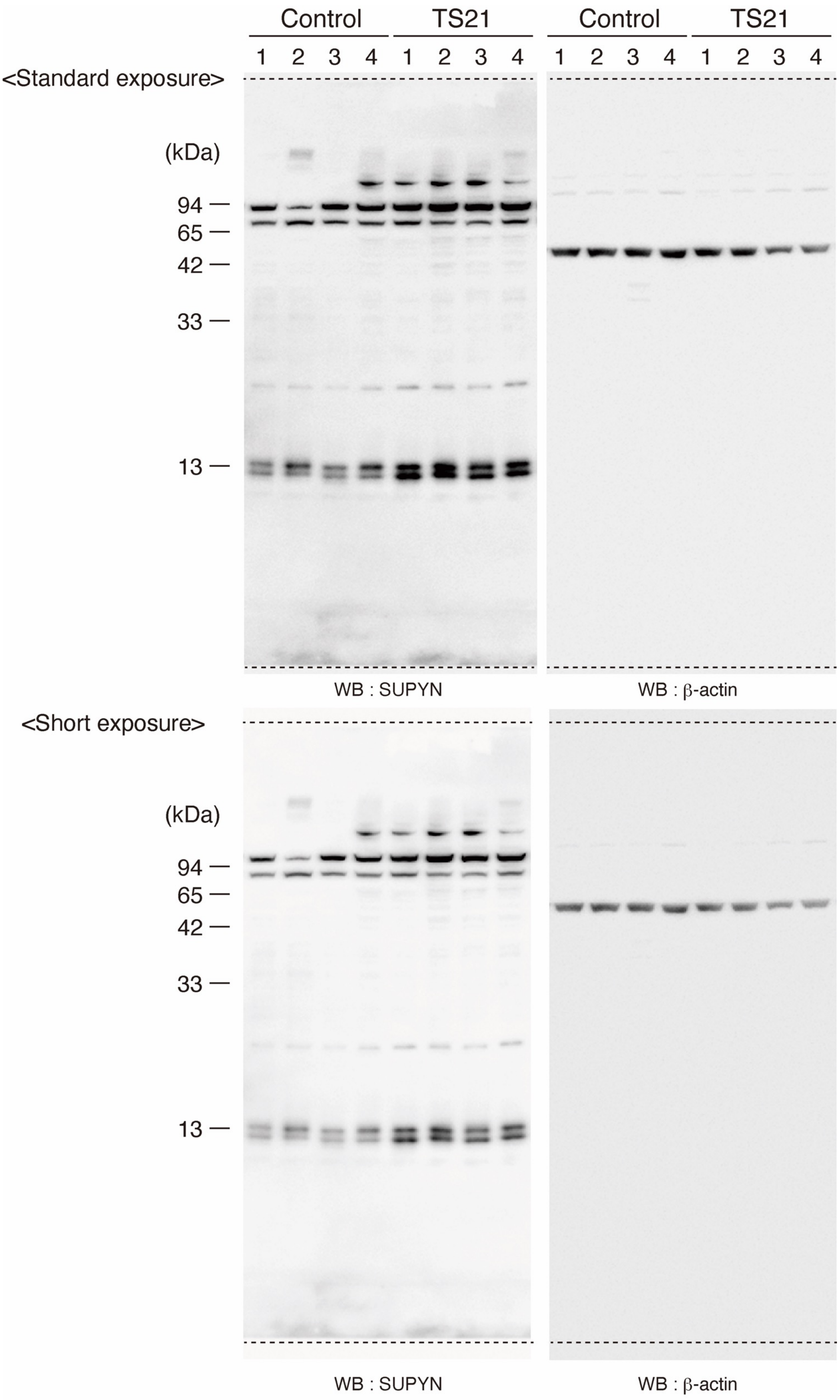

Supplementary Figure S1. Expression of *ERVH48-1* transcripts and SUPYN protein in the TS21 placenta. ; full immunoblot for Figure 1c.

Western blot analyses using a monoclonal antibody against SUPYN. Lanes 1-4: four independent human placental samples from gestational age-matched control placentas ; Lane 5-8: four independent human TS21 placentas. Standard and short exposures are included. Dotted lines indicate edge of blot.

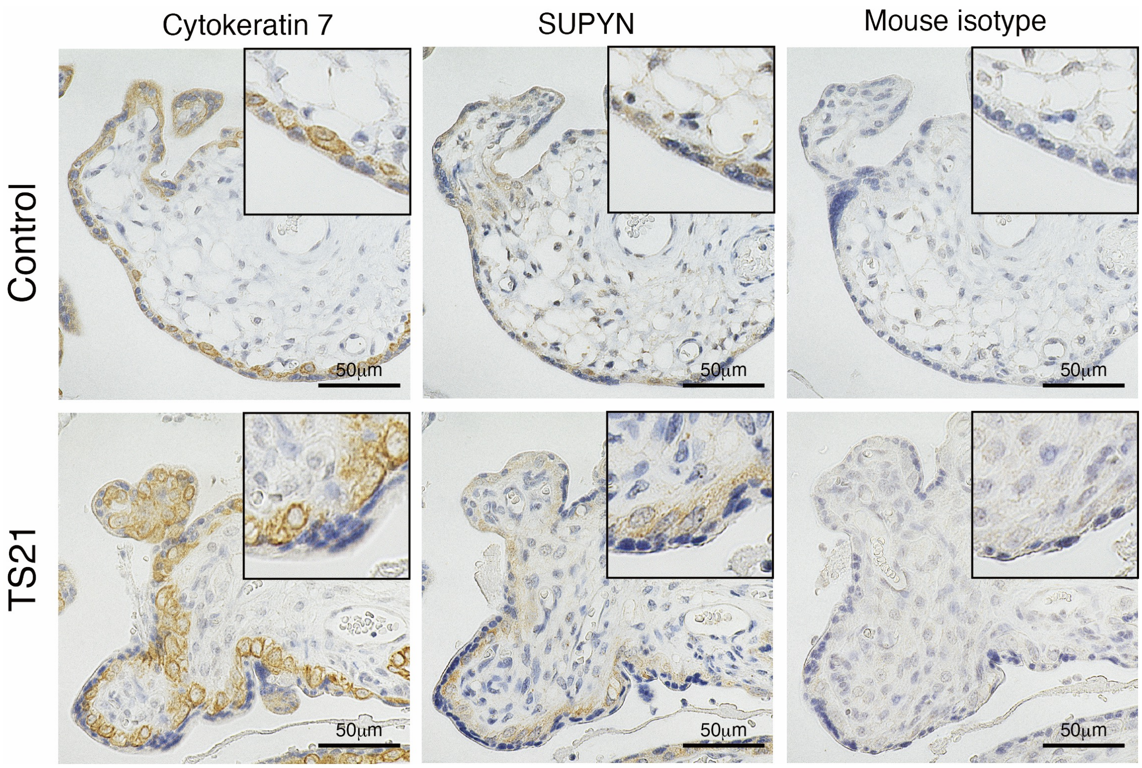

Supplementary Figure S2. Expression of SUPYN protein in the TS21 placenta. .
Immunohistochemical analysis of representative tissue samples from TS21 and gestational age-matched disomic control placentas. Immunohistochemical staining for cytokeratin 7 (left), SUPYN (middle) and mouse-isotype control (right). The upper panels are from a representative gestational age-matched control placenta, the lower panels represent a TS21 placenta. Enlargements are presented in the upper right of each panel. Scale bars indicate 50 micrometers.

a

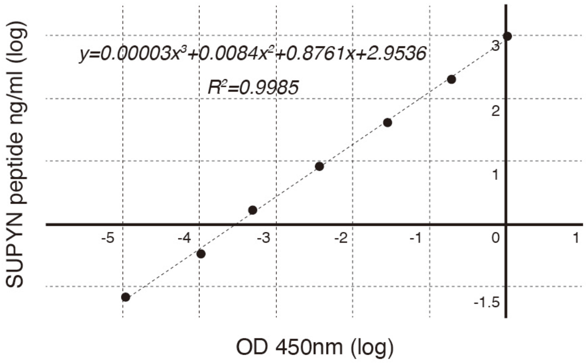

b

Spike concentration (ng/ml)	Recovery (%)
2.5	80 ± 2.2
1.25	82 ± 2.9
0.625	101 ± 4.3

Supplementary Figure S3. A SUPYN-specific ELISA was developed using two anti-SUPYN monoclonal antibodies.

a) Dilution linearity validation testing was performed using a synthetic 50aa SUPYN peptide (111-160) from 0.3125 to 20 ng/ml concentrations. SUPYN Immunoassays typically have CV values less than 10% across the standard curve for both intra- and inter-assay precision.

b) ELISA specificity was confirmed using spike assays. Purified SUPYN peptide was spiked at three concentrations into maternal serum samples. Serum background was subtracted from the spiked values and the average percent recovery is presented. These results indicate that maternal serum exerts negligible interference in the SUPYN specific ELISA.

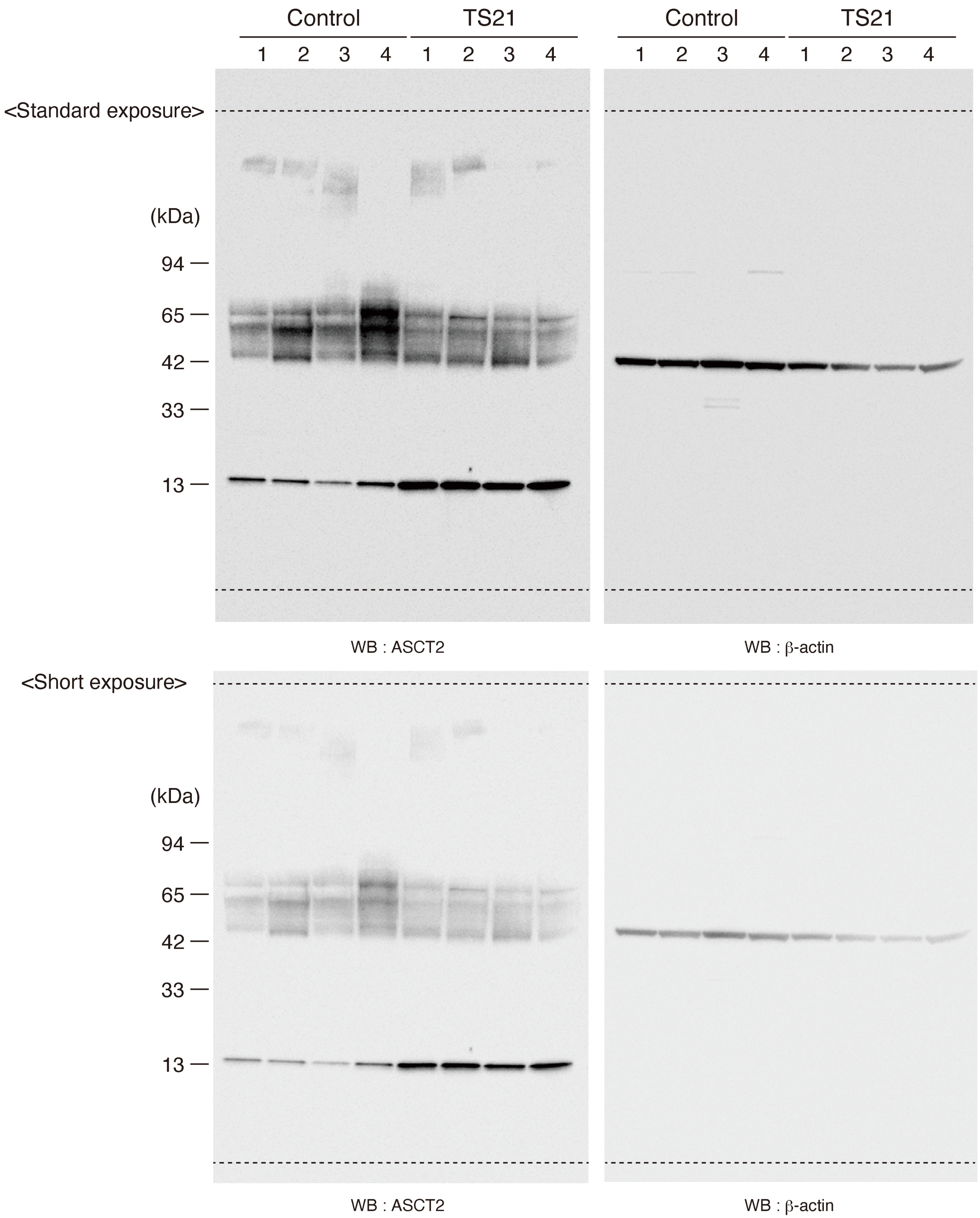

Supplementary Figure S4. Association between SUPYN and ASCT2 induces glycosylation changes in TS21 placentas. ; full immunoblot for Figure 3a.

ASCT2 western blot analysis using gestational age-matched control and TS21 placental samples. The numbers 1-4 represent sample replicates. Standard and short exposures are included. Dotted lines indicate edge of blot.

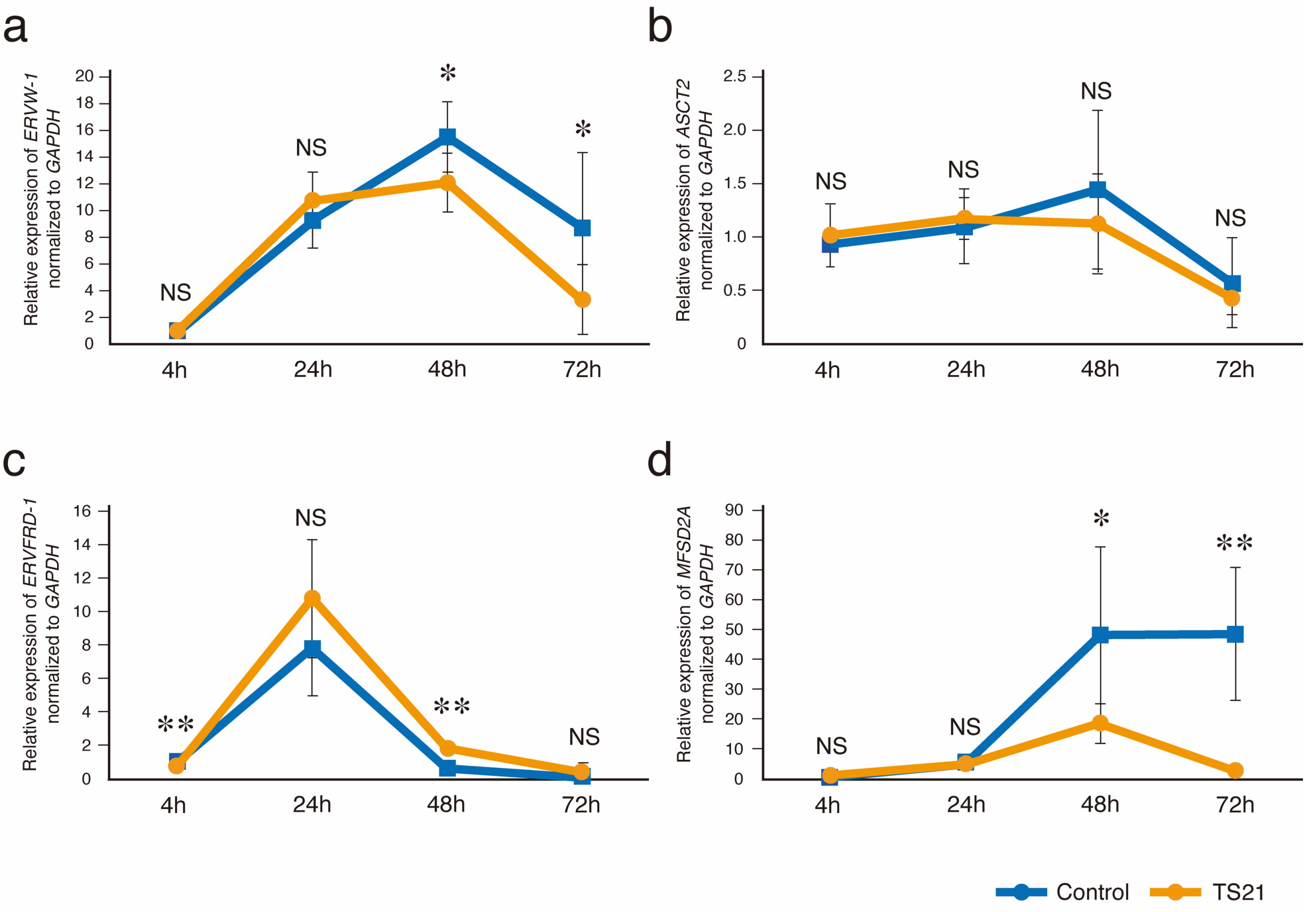

Supplementary Figure S5. Temporal expression of fusion-associated genes using quantitative RT-PCR analyses in primary trophoblast cells from TS21 and gestational matched control placentas.

Quantitative RT-PCR analyses were performed with samples from 3 independent placentas. Data were analyzed by $2^{-\Delta\Delta CT}$ methods and plotted relative fold-change compared with the normal sample at the 4h time point and normalized by expression of the *GAPDH* gene. Values represent means ± SDs (n=3). Statistical analysis was performed using the Mann Whitney U-test. A *p* value below 0.05 (* : $p<0.05$) or 0.01 (** : $p<0.01$) was considered significantly different compared with the control value at each time point. a) *ERVW-1*, b) *ASCT2* : Alanine, Serine, Cysteine Transporter 2 , the syncytin-1 receptor; c) *ERVFRD-1*, d) *MFSD2A* : major facilitator superfamily domain-containing protein 2, the syncytin-2 receptor.
